# A Randomized, Double-Blind, Parallel Study to Evaluate the Dose-Response of Three Different Vitamin D Treatment Schemes on the 25-Hydroxyvitamin D Serum Concentration in Patients with Vitamin D Deficiency

**DOI:** 10.3390/nu7075227

**Published:** 2015-07-03

**Authors:** Marie-Louise Schleck, Jean-Claude Souberbielle, Bernard Jandrain, Stéphanie Da Silva, Sophie De Niet, Francis Vanderbist, André Scheen, Etienne Cavalier

**Affiliations:** 1 Department of Clinical Chemistry, University of Liège, CHU Sart-Tilman, Liège B-4000, Belgium; E-Mail: mlschleck@chu.ulg.ac.be; 2 Laboratoire d’Explorations fonctionnelles, Hôpital Necker-Enfants malades, Paris 75015, France; E-Mail: jean-claude.souberbielle@nck.aphp.fr; 3 Department of Clinical Pharmacology, ATC SA, Liège B-4000, Belgium; E-Mail: Bernard.jandrain@atc-pharma.com; 4 Clinical Department, Laboratoires SMB SA, Brussels 1080, Belgium; E-Mails: sdsil@smb.be (S.D.S.); sdeni@smb.be (S.D.N.); fvdbi@smb.be (F.V.); 5 Division of Diabetes, Nutrition and Metabolic Disorders, CHU Sart Tilman, University of Liège, Liège B-4000, Belgium; E-Mail: andre.scheen@chu.ulg.ac.be

**Keywords:** vitamin D, randomized double-blind trial, safety

## Abstract

Many people worldwide are vitamin D (VTD) deficient or insufficient, and there is still no consensus on the dose of VTD that should be administered to achieve a 25(OH)D concentration of 20 or 30 ng/mL. In this study, we aimed to determine an adapted supplementation of VTD able to quickly and safely increase the vitamin D status of healthy adults with low 25(OH)D. One hundred and fifty (150) subjects were randomized into three groups, each to receive, orally, a loading dose of 50,000, 100,000 or 200,000 IU of VTD3 at Week 0, followed by 25,000, 50,000 or 100,000 IU at Week 4 and Week 8. Whereas 25(OH)D baseline values were not different between groups (*p* = 0.42), a significant increase was observed at Week 12 (*p* < 0.0001) with a mean change from baseline of 7.72 ± 5.08, 13.3 ± 5.88 and 20.12 ± 7.79 ng/mL. A plateau was reached after eight weeks. No related adverse event was recorded. This study demonstrated a linear dose-response relationship with an increase in 25(OH)D levels proportional to the dose administered. In conclusion, a loading dose of 200,000 IU VTD3 followed by a monthly dose of 100,000 IU is the best dosing schedule to quickly and safely correct the VTD status.

## 1. Introduction

It is estimated that one billion people worldwide are vitamin D (VTD) deficient or insufficient [1]. Indeed, VTD is found naturally in very few foods, namely pelagic fish, egg yolk, and rare plants or mushrooms. Consequently, most humans rely on endogenous synthesis of VTD, attained through exposure of the skin to ultraviolet B (UVB) light. The current consensus is to use serum 25-hydroxyvitamin D (25(OH)D) levels to estimate the VTD status, but the 25(OH)D concentration defining VTD sufficiency remains hotly debated. Indeed the Institute of Medicine (IOM) in the U.S. proposes a 25(OH)D level of 20 ng/mL as being sufficient and recommended dietary intake (RDIs) of 600 to 800 IU per day for the general population [2], whereas the Endocrine Society, whose guidelines are mainly targeted to patient care, considers that VTD deficiency corresponds to 25(OH)D levels <20 ng/mL and insufficiency to levels of 20–30 ng/mL, suggesting that a higher intake than that recommended by the IOM is necessary to obtain an optimal VTD status [3].

The amount of UVB exposure needed to achieve adequate VTD status depends on many factors such as latitude, altitude or season. In Belgium, due to the Northern latitude of the country (51° N), UVB rays are effective to allow skin synthesis of VTD during approximately only six months of the year. Accordingly, a large part of the Belgian population has VTD deficiency, at least during the less sunny months, even when considering the most conservative level of 20 ng/mL used to define deficiency [2]. As usual daily intakes are generally not higher than 200 IU in western countries [4,5], a small increase in the amount of supplementation is probably needed in most Belgian healthy individuals in order to achieve the IOM goals. In some patient groups, such as those with osteoporosis, end-stage renal disease, primary hyperparathyroidism, malabsorption, or frail elderly patients at risk of falls, a significantly higher amount of VTD will be needed to achieve the target level of 30 ng/mL, a threshold above which positive health outcomes have been observed [6,7,8]. In current clinical practice, it is usual to prescribe initially high loading doses in those individuals with a low serum 25(OH)D concentration in order to reach the desired target level (20 or 30 ng/mL depending on the individual patient), and to continue with long-term treatment using lower doses in order to maintain the 25(OH)D level above the target [1,9]. Unfortunately, the precise dosing scheme allowing all these patients to reach rapidly and safely a 25(OH)D level above 20 or 30 ng/mL still remains to be determined. Furthermore, the pharmaceutical vitamin D supplements that are currently available differ greatly from one country to another, making a universal scheme of supplementation hard to define. In Belgium, D-CURE^®^ (Laboratoires SMB), an oily solution containing 25,000 IU of cholecalciferol (VTD3) per ampoule for oral use, is one of the major pharmaceutical VTD supplements. In a previous randomized placebo controlled trial, a supplementation scheme with D-CURE^®^ based on four models according to the baseline 25(OH)D concentrations of the subjects was tested. While a significant change in 25(OH)D compared to placebo was observed, values ≥30 ng/mL were only achieved in 57.1% of subjects receiving VTD3, indicating that the doses may need to be increased in subsequent studies [10].

In this new randomized, double-blind, parallel study, the objective was to determine if an adapted supplementation of VTD3 (D-CURE^®^) is able to quickly and safely increase the vitamin D status of patients with a low 25(OH)D concentration and to see how many healthy subjects would increase their 25(OH)D levels above 20 ng/mL and 30 ng/mL, as suggested by the IOM or the Endocrine Society, respectively.

## 2. Methods

### 2.1. Methodology

The study took place between the 12 December, 2012 and 3 May, 2013. The study recruitment was performed in one Belgian site in the area of Liège by independent investigators. One hundred and ninety-six (196) healthy adults with low 25(OH)D were screened within 28 days prior to the start of the study. Volunteers were initially screened via telephone and again on attendance at the screening visit.

Those who met all of the inclusion and none of the exclusion criteria were randomly assigned to one of three different treatment groups, each with a different VTD3 dosing scheme. The subjects took the study medication at one monthly intervals under the supervision of the study personnel at Visit 2 (week 0), Visit 3 (week 4) and Visit 4 (week 8). The total duration of the study was 12 weeks with an 8-week period of supplementation followed by a 4-week period without supplementation until the last blood sample was drawn.

Caucasian (defined as European and North African) male or female subjects aged more than 18 years with a 25 OH-vitamin D ≥5 ng/mL and ≤20 ngmL were included at the screening visit. Subjects presenting a body mass index (BMI) between 18 and 30 kg/m^2^, normal thyroid function confirmed by a normal TSH value with or without treatment and who were able to comply with all study procedures were included. They all gave their written, informed consent to participate in this trial.

This trial was approved by the Independent Ethics Committee (IEC) and by the Belgian Competent Authorities (Protocol No. D-CURE-IV-12-2 and EudraCT No. 2012-004917-14). This study was conducted in accordance with the ethical principles that have their origin in the Declaration of Helsinki and that are consistent with Good Clinical Practice (GCPs/ICH E6-Step 5)––including the International Conference on Harmonization (ICH) Guidelines—and the requirements according to the National Drug Law in application in Belgium in which the study was performed.

The exclusion criteria were past or current history of any immunological, neoplasic, endocrine, hematological, hepatic, renal, gastrointestinal, neurological, or psychiatric abnormalities or medical disease and pregnancy. Subjects who used a UV light solarium two weeks before the screening visit or any type of vitamin D supplement within four weeks before the screening visit or planned to travel outside European countries during the study were excluded. Patients under treatment that could potentially interfere with vitamin D metabolism and those with past or current history of granulomatosis, especially sarcoidosis, urinary lithiasis and osteomalacia were also excluded. Finally, patients who presented a serum creatinine >150 µmol/L and albumin corrected serum calcium >2.65 mmol/L (corresponding to 10.6 mg/dL) at screening were excluded, as well as those with any sensitivity or allergy to any of the products used in the study or a history of drug and/or alcohol abuse.

### 2.2. Randomization

At the screening visit, 196 patients were screened. Forty-six (46) of the 196 screened patients were excluded from the trial. The main reasons for the screening failures were a level of 25-OH vitamin D above 20 ng/mL (*n* = 15) and abnormal laboratory values (*n* = 12).

At the randomization visit (Visit 2), the selected subjects were randomized into three groups of 50, each to receive a low, moderate or high dose of D-CURE^®^ once a month. D-CURE^®^ consists of an ampoule containing an oily solution of 25,000 IU VTD3 for oral use. Three dosing schedules were compared: subjects received a loading dose of either 50,000, 100,000 or 200,000 IU of VTD3 according to their group at Week 0, followed by 25,000, 50,000 or 100,000 at Week 4 and Week 8. The total study duration was 12 weeks. A total dose of 100,000, 200,000 and 400,000 IU, respectively, was therefore administered over 12 weeks. (Table 1). Placebo ampoules were added so that the same number was taken in each treatment sequence in order to maintain the blind between the three different randomization schemes.

**Table 1:** Treatment scheme with different intakes of vitamin D (VTD3) according to randomization group.

	Treatment Period			Total Treatment Dose
	Visit 2 Week 0	Visit 3 Week 4	Visit 4 Week 8	
Group 1	2 ampoules of 25,000 IU	1 ampoule of 25,000 IU	1 ampoule of 25,000 IU	100,000 IU
*n* = 50	+ 6 ampoules of placebo	+ 3 ampoules of placebo	+ 3 ampoules of placebo	
Group 2	4 ampoules of 25,000 IU	2 ampoules of 25,000 IU	2 ampoules of 25,000 IU	200,000 IU
*n* = 50	+ 4 ampoules of placebo	+ 2 ampoules of placebo	+ 2 ampoules of placebo	
Group 3	8 ampoules of 25,000 IU	4 ampoules of 25,000 IU	4 ampoules of 25,000 IU	400,000 IU
*n* = 50				

### 2.3. Laboratory Assessment and Methods

Blood samples were obtained at screening and at the end of the study (Week 12) for safety analyses (especially haematology, chemistry, calcium, and phosphate). Additional blood samples were taken for 25(OH)D assessment at screening, Week 0, 4, 8, and 12. Blood samples were drawn just before the intake of the next dose of VTD3. The blood sample at week 12 which was four weeks after the intake of the final VTD3 dose.

The DiaSorin Liaison assay method (Stillwater, MN, USA) was used for 25(OH)D measurement. The intra-, and inter-assay coefficients of variation were <5% and <10% respectively. The functional detection limit was 4 ng/mL. Other biological laboratory values including serum calcium concentrations were evaluated with the Roche Modular instrument (Mannheim, Germany).

### 2.4. Statistical Methods

The results were computed using the raw SAS Version 9.3. Between group comparisons were performed at baseline to detect any imbalance between the three treatment groups using an ANOVA test for quantitative variables (age, BMI) and a chi^2^ test for qualitative variables (sex). The mean changes of 25(OH)D were compared between the three groups with a two-sided 98.3% confidence interval. The time to raise the 25-OH vitamin D serum concentration >20 ng/mL and >30 ng/mL was estimated with a time-to-event model (Kaplan Meier’s estimator). The percentage of patients reaching the target level at each time point was compared between groups using a chi square test.

## 3. Results

One hundred and forty-eight (148) subjects completed the entire study (50 patients from Group 1, 49 patients from Group 2, and 49 patients from Group 3). One patient from Group 2 discontinued the study after visit 4 (Week 8) due to an adverse event not related to the study treatment. One patient from Group 3 discontinued the study after visit 2 (Week 0) for lack of compliance to the protocol. The patient took only the first dose of study treatment of 200,000 IU of VTD3.

The main demographic characteristics of the subjects are presented in Table 2. As the treatment was taken under the supervision of the study personnel, compliance was 100%.

**Table 2:** Demographic data of the 150 subjects included in the study.

	Group 1	Group 2	Group 3	*p* value
	(*n* = 50)	(*n* = 50)	(*n* = 50)	
Age (years)				
Mean ± SD	27.3 ± 9.5	31.0 ± 10.1	29.3 ± 9.2	0.16
Min–max	18–53	19–57	19–53	
BMI (kg/m^2^)				
Mean ± SD	22.8 ± 2.6	23.9 ± 3.4	23.1 ± 2.9	0.17
Min–max	18–30	18–31	18–29	
25(OH)D				
Mean ± SD	14.5 ± 3.5	13.5 ± 3.7	14.2 ± 3.8	0.4
Min–max	7–20	5–20	6–20	

According to the protocol requirements, all patients had a 25(OH)D level between 5 and 20 ng/mL at the screening visit without any significant difference between randomization groups (*p* = 0.40, ANOVA test). The level of 25(OH)D remained relatively unchanged throughout the screening period up to the randomization visit (mean baseline level: 13.53 ± 3.72 ng/mL).

### 3.1. Mean Change of 25(OH)D from Baseline to Week 12

Whereas baseline values were not different between groups (*p* = 0.42), a significant increase was observed between groups after the 12-week treatment period (*p* < 0.0001) with a mean change from baseline of 7.72 ± 5.08, 13.3 ± 5.88 and 20.12 ± 7.79 ng/mL for Group 1, Group 2, and Group 3 respectively. The mean change of 25(OH)D serum concentration at each time point is shown in Table 3 for each group.

**Table 3:** Mean change of 25(OH)D (ng/mL) serum concentrations over time.

Mean Change in 25(OH)D level (ng/mL)		From baseline to week 4	From baseline to week 8	From baseline to week 12
	Group 1	6.12 ± 4.82	7.98 ± 5.06	7.72 ± 5.08
	*n* = 50	−3–18	–3–20	−1–18
Mean ± SD	Group 2	9.08 ± 5.74	13.36 ± 6.21	13.30 ± 5.88
Min–Max	*n* = 50	−2–22	4–26	4–27
	Group 3	15.64 ± 7.38	19.80 ± 7.88	20.12 ± 7.79
	*n* = 50	2–33	3–47	0–43
*p* value		<0.0001	<0.0001	<0.0001

### 3.2. Evolution of 25(OH)D Serum Concentrations over Time

**Figure 1:**
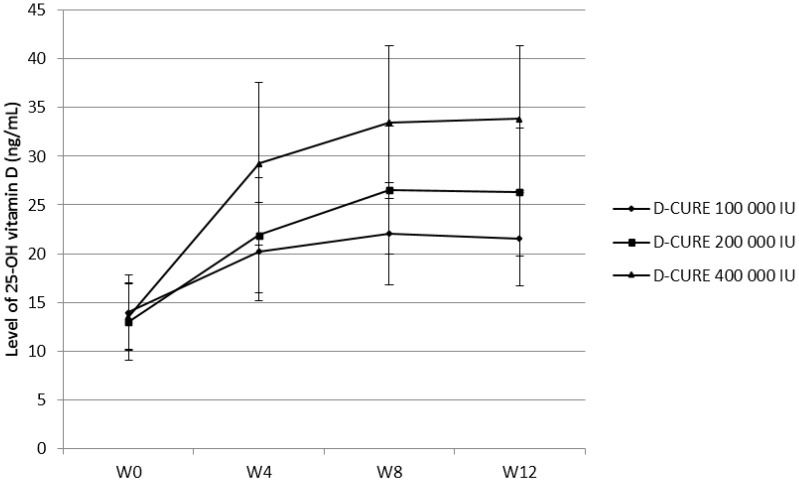
Evolution of 25(OH)D (ng/mL) over time.

The 25(OH)D serum concentrations significantly increased over time and reached a plateau at Week 8 (Figure 1). We observed higher increases in 25(OH)D levels for the higher doses ofD-CURE^®^. Hence, the difference in 25(OH)D serum concentrations between treatment groups was statistically significant at each time point following the baseline (*p* < 0.0001).

There were no significant changes from the baseline at week 12 seen in the mean plasma calcium concentration during the study (−0.08 ± 0.10 mmol/L) (Table 4). There were no cases of hypercalcemia or nephrolithiasis that occurred during the study. No adverse events related to the study treatment and no changes in hematological and biochemistry laboratory values were detected.

**Table 4:** Evolution of 25(OH)D (ng/mL) and calcium (mmol/L) serum concentrations over time.

		Visit 1	Visit 2	Visit 3	Visit 4	Visit 5
		Screening	Week 0	Week 4	Week 8	Week 12
	25(OH)D level	14.52 ± 3.47	13.94 ± 3.75	20.06 ± 5.05	21.92 ± 5.22	21.66 ± 4.95
	m ± SD (ng/mL)					
**Group 1**	Min-Max	7.0–20.0	7.0–22.0	9.0–30.0	11.0–32.0	13.0–33.0
*n* = 50	Calcium level	2.48 ± 0.08				2.39 ± 0.08
	m ± SD (mmol/l)					
	Min–Max	2.28–2.63				2.25–2.57
	25(OH)D level	13.55 ± 3.71	12.98 ± 3.85	22.06 ± 5.93	26.34 ± 6.52	26.28 ± 6.45
	m ± SD (ng/mL)					
**Group 2**	Min-Max	5.0–20	6.0–21.0	7.0–33.0	11.0–37.0	11.0–38.0
*n* = 50	Calcium level	2.45 ± 0.1				2.36 ± 0.09
	m ± SD (mmol/l)					
	Min–Max	2.21–2.62				2.19–2.56
	25(OH)D level	14.25 ± 3.76	13.66 ± 3.57	29.3 ± 8.26	33.46 ± 7.78	33.78 ± 7.51
	m ± SD (ng/mL)					
**Group 3**	Min-Max	6.1–20.0	7.0–20.0	13.0–50.0	17.0–64.0	14.0–60.0
*n* = 50	Calcium level	2.44 ± 0.09				2.38 ± 0.08
	m ± SD (mmol/l)					
	Min–Max	2.14–2.61				2.16–2.55

### 3.3. Percentage of Patients Reaching a 20 ng/mL or 30 ng/mL 25(OH)D Serum Concentration over Time, and Time to Raise 25(OH)D Serum Concentration to 20 or 30 ng/mL

At the end of the study (Week 12), 78% (*n* = 117) and 30.7% (*n* = 46) of the subjects reached a 25(OH)D serum concentration >20 ng/mL and >30 ng/mL respectively. Time to event (20 or 30 ng/mL) was different between groups and shorter for higher doses of D-CURE^®^.

Regardless of the visit, the proportion of patients achieving the 20 ng/mL or 30 ng/mL target level was significantly different between groups. The number of subjects that reached 25(OH)D levels of 20 ng/mL or 30 ng/mL at each time point is shown in Table 5 for each treated group separately. The highest number of subjects that did reach 25(OH)D serum concentrations of 30 ng/mL at week 8 was seen in Group 3 (*n* = 62%). There were 6% of the subjects and 30% of the subjects who did reach serum concentrations of 25(OH)D>30 ng/mL at week 8 in Groups 1 and 2, respectively. Likewise, 98% of the subjects from Group 3 reached the target level of 20 ng/mL at week 8, whereas only 58% and 78% of the subjects from Group 1 and Group 2 respectively reached this target at week 12.

**Table 5:** Number (%) of subjects who reached the target levels of 20 ng/mL and 30 ng/mL at all time points.

	Group 1 *n* = 50	Group 2 *n* = 50	Group 3 *n* = 50	*p* value
25(OH)D >20 ng/mL				
Target reached at W4	23 (46.00%)	34 (68.00%)	43 (86.00%)	0.0001
Target reached at W8	29 (58.00%)	39 (78.00%)	49 (98.00%)	<0.0001
Target reached at W12	26 (52.00%)	42 (84.00%)	49 (98.00%)	<0.0001
25(OH)D >30 ng/mL				
Target reached at W4	0 (0.00%)	3 (6.00%)	21 (42.00%)	<0.0001
Target reached at W8	3 (6.00%)	15 (30.00%)	31 (62.00%)	<0.0001
Target reached at W12	2 (4.00%)	12 (24.00%)	32 (64.00%)	<0.0001

## 4. Discussion

Many people worldwide are VTD deficient or insufficient, and there is still no consensus on the dose of vitamin D that should be administered to achieve a 20 or 30 ng/mL vitamin D concentration.

This study was performed to compare three different VTD3 dosing schedules in their ability to increase the 25(OH)D concentration above 20 or 30 ng/mL in subjects who were initially VTD-deficient (≤20 ng/mL).

At week 12 of the study, one month after the two month supplementation period with doses of VTD3 corresponding roughly to 1667 to 6667 IU/day, the mean change from the baseline in the different subgroups ranged from +7.7 to +20.1 ng/mL corresponding to 0.30 to 0.46 ng/mL for 100 IU. This is quite similar to an increase of 0.72 ng/mL for 100 IU reported by Heaney [11], but much less than that found by Autier [12] or McKenna [13] who reported in their meta-analyses of interventional studies with different doses of VTD, an increase of 1.96 ng/mL and 2.12 ng/mL for 100 IU vitamin D respectively. It must be noted that in [12,13], the studies that were analyzed most used daily administration of VTD. In the present study, instead of daily doses, loading doses were given in the presence of the study personnel to optimize observance to supplementation. The measurement of the 25(OH)D concentration was also performed one month after each dose. As the 25(OH)D peak concentration after quite a large dose usually occurs within one week after administration and decreases thereafter [14,15], it is likely that, in the present study, the 25(OH)D concentration had already decreased at the moment that the blood samples were obtained, explaining in part the lower increase in the 25(OH)D concentration than in other studies [12,13].

A serum concentration of 25(OH)D >20 ng/mL was obtained for almost all subjects (98%) as early as week 8 in the 400,000 IU VTD3 supplemented group), while 84% and 52% of patients in the 20,000 IU and 100,000 IU VTD3 supplemented groups respectively attained this target only after 12 weeks of treatment. Likewise, more patients attained the target of 30 ng/mL at week 12 in the 400,000 IU VTD3 group (64%) compared to the lower dosage supplemented groups (24% and 4% for the 200,000 IU and 100,000 IU VTD3 supplemented groups, respectively).

These supplementations were demonstrated to be safe as no variation in calcium levels and no clinically relevant adverse events between the groups after three months of supplementation with VTD3 were observed, even with the higher doses. The highest value of 25(OH)D achieved with this protocol was 64 ng/mL at week 8 in a patient in the 400,000 IU VTD3 supplemented group (Table 4).

The main strengths of this study are its double-blind, placebo-controlled design, allowing to control for possible confounders such as season-, or diet-related changes in the 25(OH)D level, and the supervision by the study staff of the administration of all VTD3 doses allowing for a compliance of 100%. Indeed, as the study took place during winter, and as none of the subjects were exposed to tropical or high-altitude sunshine, this suggests that the observed increases in 25(OH)D concentrations were only due to the treatment alone. The study has also some limitations. As the supplements were given under controlled conditions, it should be verified that they can also apply to different categories of patients. In particular, as the subjects included in the study were generally not overweight (BMI 23 kg/m^2^), and as obese people need more VTD3 than those of normal-weight in order to attain similar levels of 25(OH)D, another study using the same supplementation schemes should be initiated in patients with a BMI >30 kg/m^2^.

## 5. Conclusions

In conclusion, this study demonstrated a dose-response relationship presenting a logarithmic pattern between the three patient groups, with an increase in 25(OH)D levels proportional to the dose administered and a plateau after four weeks. The higher the dosing of VTD3, the greater the change in the 25(OH)D serum concentration. A loading dose of 200,000 IU VTD3 followed by a monthly dose of 100,000 IU is, among the doses tested in this study, the best one to quickly and safely correct the VTD status.
